# Dabigatran in Secondary Stroke Prevention: Clinical Experience with 106 Patients

**DOI:** 10.1155/2014/567026

**Published:** 2014-07-15

**Authors:** Alicia DeFelipe-Mimbrera, Araceli Alonso Cánovas, Marta Guillán, Consuelo Matute, Susana Sainz de la Maza, Antonio Cruz, Rocío Vera, Jaime Masjuan

**Affiliations:** Stroke Unit, Neurology Department, IRYCIS, University Hospital Ramón y Cajal, Carretera de Colmenar Viejo km 9,100, 28034 Madrid, Spain

## Abstract

*Introduction*. Our aim was to analyze our clinical experience with dabigatran etexilate in secondary stroke prevention. *Methods*. We retrospectively included patients starting dabigatran etexilate for secondary stroke prevention from March 2010 to December 2012. Efficacy and safety variables were registered. *Results*. 106 patients were included, median follow-up of 12 months (range 1–31). Fifty-six females (52.8%), mean age 76.4 (range 50–95, SD 9.8), median CHADS2 4 (range 2–6), CHA2DS2-VASc 5 (range 2–9), and HAS-BLED 2 (range 1–5). Indication for dabigatran etexilate was ischemic stroke in 101 patients and acute cerebral hemorrhage (CH) due to warfarin in 5 (4.7%). Dabigatran etexilate 110 mg bid was prescribed in 71 cases (67%) and 150 mg bid was prescribed in the remaining. Seventeen patients (16%) suffered 20 complications during follow-up. Ischemic complications (10) were 6 transient ischemic attacks (TIA), 3 ischemic strokes, and 1 acute coronary syndrome. Hemorrhagic complications (10) were CH (1), gastrointestinal bleeding (6), mild hematuria (2), and mild metrorrhagia (1), leading to dabigatran etexilate discontinuation in 3 patients. Patients with previous CH remained uneventful. Three patients died (pneumonia, congestive heart failure, and acute cholecystitis) and 9 were lost during follow-up. *Conclusions*. Dabigatran etexilate was safe and effective in secondary stroke prevention in clinical practice, including a small number of patients with previous history of CH.

## 1. Introduction

Ischemic stroke is one of the most common complications of atrial fibrillation (AF). The mean annual rate for ischemic stroke in patients with nonvalvular AF (NVAF) is 5%, rising to 23% in patients over 80 years old [1, 2].

Vitamin K antagonists (VKA) have been shown to be effective in reducing the incidence of stroke, even in the very elderly [3], and in secondary stroke prevention [4]. However, significant limitations such as individual variability in pharmacokinetics, the need for monitoring, interactions with both drugs and foods, and the risk of bleeding have led to the development of the new oral anticoagulants (NOAC) [5].

One of these NOACs is dabigatran etexilate, a direct thrombin inhibitor approved by the European Medicines Agency in August 2011, based on the results of the clinical trial entitled “Randomized Evaluation of Long-Term Anticoagulation Therapy (RE-LY)” [6], for primary and secondary prevention of cardioembolic stroke in patients with NVAF [7]. In the direct comparison with warfarin, dabigatran etexilate 150 mg bid (D150) was superior in preventing stroke and systemic embolism, with a 24% relative risk reduction (RRR) for stroke, while the 110 mg bid dose (D110) was shown to be noninferior to warfarin and safer [6]. Furthermore, both doses resulted in a lower risk of cerebral hemorrhage (CH) compared to warfarin, even in patients over 75 years old [5, 6, 8, 9]. A subgroup analysis in secondary stroke prevention with 3,623 patients showed a nonsignificant trend in favor of dabigatran etexilate having greater efficacy, with a significantly lower rate of CH [10]. Dabigatran etexilate has also been shown in postmarketing surveillance studies to be both safe [11] and cost-effective [12]. Clinical guidelines recommend the use of dabigatran etexilate over VKA in primary and secondary prevention of stroke in patients with NVAF, in case of the AHA guidelines with a level of evidence B [13–16]. The most effective dose of dabigatran etexilate is 150 mg/12 hours, although 110 mg/12 hours should be used in certain circumstances (over 75 years old, creatinine clearance 30–50 mL/min, patients at high risk of hemorrhage (HAS-BLED ≥ 3) and in patients treated with verapamil, as recommended by the European Union guidelines [8, 17, 18]. In the United States the Food and Drug Administration approved dabigatran etexilate 75 mg bid instead of 110 mg bid.

Despite the above, there is a lack of quality efficacy and safety data on the use of dabigatran etexilate in secondary prevention of stroke in routine clinical practice [19]. Moreover, ischemic and hemorrhagic risk in the patients selected for the clinical trials may be lower than in actual clinical practice. Data regarding efficacy and safety in unselected populations is lacking, although at least two observational registries are ongoing, and the FDA reported data regarding safety in clinical practice [20–23]. Our aim was to analyze our clinical experience with dabigatran etexilate in secondary stroke prevention.

## 2. Methods

This was a retrospective analysis of patients treated with dabigatran etexilate for secondary prevention of stroke at our large teaching hospital between March, 1, 2010 and December, 31, 2012. Independent Ethics Committee approval was obtained.

Criteria for starting treatment with dabigatran etexilate were diagnosis of stroke or TIA secondary to NVAF, no absolute contraindications for anticoagulation, normal liver function, and glomerular filtration rate (GFR) ≥ 30 mL/min.

Demographic and clinical variables, history of treatment with VKA, and analytical data (INR, GFR) were all recorded. The stroke or systemic embolism and hemorrhage risks were assessed using the CHADS2, CHA2DS2-VASc, and HAS-BLED scores. The dabigatran etexilate dose and the concomitant use of antiaggregation therapy were specified.

Patients were followed up through outpatient appointments and review of electronic records until their death or loss to follow up. Observation time (months), ischemic and hemorrhagic complications, and time to the event (months) were recorded. During follow-up, adherence to dabigatran etexilate was assessed by means of clinical questioning. CH and any bleeding with anemia, considered as 2-point drop in hemoglobin measured in g/dL, and/or need for red blood cell transfusion were considered serious adverse events, following the definition of RE-LY of major bleeding [6].

*Statistical Methods*. We performed a descriptive analysis of baseline clinical variables (gender, age > 75, hypertension, DM, reason for starting dabigatran etexilate, previous anticoagulation, CHA2DS2-VASc > 5, GFR < 60, dabigatran etexilate dose, concomitant antiaggregation therapy). The data were analyzed using SPSS 20 statistical software. Categorical variables are presented as absolute numbers and frequencies and quantitative variables are expressed as mean (standard deviation) and median (min-max range). Treatment continuation is represented by Kaplan-Meier survival curve.

## 3. Results

**Figure 1:**
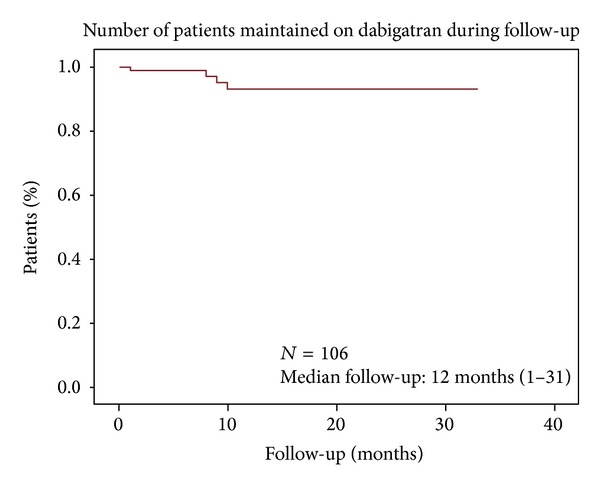
Number of patients maintained on dabigatran during follow-up (Kaplan-Meier survival curve).

106 patients were included with a median follow-up of 12 months (range 1–33). 56 were female (52.8%), with a mean age 76.4 years (range 50–95, SD 9.8), median CHADS2 4 (range 2–6), CHA2DS2-VASc 5 (range 2–9), and HAS-BLED 2 (range 1–5). Indication for dabigatran etexilate was ischemic stroke in 101 patients (66.3% anticoagulation naïve and 33.7% previously on warfarin) and acute CH due to VKA in 5 (4.7%). Dabigatran etexilate 110 mg bid was prescribed in 71 cases (67%), mostly due to age over 75 years (Table 1), and 150 mg bid was prescribed in the remaining. Adherence was confirmed for every patient. Sixty-six patients who suffered ischemic stroke were previously diagnosed as NVAF, only 34 (51.5%) were on VKA, and 29 (85,3%) had INR below 2. All patients with CH due to VKA had INR above 3 at the time of diagnosis. Nine patients received concomitant antiaggregation therapy due to vascular disease. Baseline characteristics, treatment indication, and concomitant medication are summarized in Table 1. Seventeen patients (16%) suffered 20 events during follow-up and 3 patients suffered 2 events. Ischemic complications (10) (Table 2) consisted of 6 transient ischemic attacks (TIAs), 3 ischemic strokes (2 of them disabling), 1 treated with intravenous thrombolysis, and 1 acute coronary syndrome. Three TIAs and all ischemic strokes occurred during the first month of treatment. Hemorrhagic complications (10) (Table 3) were CH (1, not disabling), gastrointestinal bleeding (6, of which 3 required blood transfusions), mild hematuria (2), and mild metrorrhagia (1). Four patients suffered serious adverse events. Events only led to dabigatran etexilate discontinuation in 4 patients (Figure 1).

All patients treated with dabigatran etexilate because they had a previous CH due to VKA remained uneventful. Three patients died due to pneumonia, congestive heart failure, and acute cholecystitis and 9 were lost during follow-up.

## 4. Discussion

We present a series of patients treated with dabigatran etexilate for secondary prevention of stroke with reasonably favourable results in terms of efficacy and safety, taking into account the high-risk features of our patients. The prevention of stroke in patients with NVAF is a serious problem which is far from being resolved. A study of NVAF patients admitted for a first stroke found that 60% were not receiving anticoagulation and of those taking VKA, 75% had subtherapeutic INR [24]. Our data are similar in that only 34 of the 66 patients with a history of NVAF with ischemic stroke were treated with VKA, and 29 (85,3%) of them had a subtherapeutic INR. These results reflect the continued underuse of anticoagulant therapy, despite the clinical guideline recommendations and the difficulty in maintaining the INR in the therapeutic range in patients treated with VKA. This phenomenon is not considered to be explained by suboptimal adherence, but for VKA erratic pharmacokinetics [24].

Our patients treated with dabigatran etexilate had a higher ischemic and hemorrhagic risk profile than the patients included in the RE-LY study [6, 25]. The mean age was higher (76 versus 71 years) and there was a larger proportion of women (52.8% versus 36.4%). In addition, all had previously suffered a stroke or TIA, as opposed to only 20% of the RE-LY patients. The RE-LY secondary-prevention subgroup had a similar CHADS2 score to our cohort but with a lower age (mean 70.5 years) [10]. Moreover, we included 5 patients with CH, which was an exclusion criterion in RE-LY. In our series, a majority (67%) received D110, mainly, because they were aged > 75 and due to their high hemorrhagic risk.

During follow-up, a total of 20 adverse events were recorded in 17 patients, 10 ischemic (9.4%) (Table 2) and 10 hemorrhagic (9.4%) (Table 3). The mortality rate was low (2.8%) and unrelated to dabigatran etexilate, despite the fact that the patients were elderly and suffered from multiple medical problems. It is also worth noting that treatment only had to be permanently discontinued in 4 patients (Figure 1).

The RE-LY study provided us with an estimated annual risk of stroke in patients on treatment with dabigatran etexilate of around 1% in primary prevention and 2% in secondary prevention [6, 10]. Although our event rate may seem higher (around 10%), the higher risk basal features of our cohort may be accounted for this increase [25]. In addition, if only cerebral infarcts are considered, we observed 3 cases (2 of them disabling), a figure consistent with RE-LY study. The majority of cerebral ischemic events occurred during the first month of treatment, which might suggest that this is a high-risk period. In terms of other ischemic events, in our series, a patient with a history of revascularized chronic ischemic heart disease suffered an acute coronary syndrome which required stenting.

Recently, a Danish registry of dabigatran etexilate in clinical practice and a FDA report on dabigatran etexilate safety confirm a bleeding risk of this drug comparable to warfarine, with lower CH rate [20, 23]. Moreover, Weber effect (an increased likelihood of reporting adverse events in newly approved drugs) may account for the excess of reports of bleeding complications in patients treated with dabigatran etexilate [23]. In our cohort there were 9 systemic bleeding complications, none of which were fatal, and only 3 required blood transfusions. Only in 3 cases dabigatran etexilate had to be discontinued permanently. The treatment for the bleeding episodes consisted of temporarily discontinuing dabigatran etexilate. None of the patients required prothrombin complex concentrates or hemodialysis. Although there is considered to be a lower risk of bleeding with D110, [5, 6] in our series, the majority of patients with hemorrhagic complications were taking this dose. Therefore, despite D110 seeming to be the best option in patients at high risk of bleeding, clinical and laboratory data should be monitored for signs of such events. Several factors such as age, renal function impairment, and comedications increase the risk of hemorrhagic complications [26]. The concomitant use of antiaggregation therapy increased the risk of major bleeding in the RE-LY patients [27]. In our series, of the 9 patients treated with dual therapy, 2 had hemorrhagic complications; although neither was serious, in one case dabigatran etexilate was discontinued permanently for safety concerns. These data suggest that the concomitant use of antiaggregation therapy may be safe, but further evidence is required to confirm this and these patients need to be closely monitored for bleeding events.

The most feared complication of anticoagulant treatment is CH. The risk is however lower with either dose of dabigatran etexilate than with warfarin [10]. In our series, there was only 1 CH during the follow-up period, with the 110 mg dose. None of the patients on dual therapy or with a history of previous VKA-related CH suffered a CH. The reasons for the lower rate of intracranial bleeding with either dose of dabigatran etexilate are not fully understood. It is speculated that dabigatran etexilate's single therapeutic target could preserve certain hemostatic mechanisms in the brain that may be protective against spontaneous CH [8]. However, ongoing registries will help clarify safety of dabigatran etexilate in routine clinical practice [21, 22].

Our study had a high percentage of anticoagulation-naïve patients (63.2%), slightly higher than that in the RE-LY study (50.4%). As in RE-LY, their outcome did not differ significantly from that of the rest of the patients [28, 29]. Dabigatran etexilate therefore appears to be a safe and effective alternative for patients who have not previously received VKA.

Our study has some limitations, such as the limited number of patients and the short follow-up period in a proportion of cases. Nevertheless, these were 106 patients with high baseline risk treated with dabigatran etexilate for secondary stroke prevention with a satisfactory overall outcome, with high treatment adherence and a relatively low rate of severe or disabling events. These data confirm in routine clinical practice the published benefits of dabigatran etexilate in secondary stroke prevention found in controlled clinical trials. However, caution is needed, as this treatment is not exempt of complications, as our data show.

## Figures and Tables

**Table 1:** Baseline characteristics.

Baseline clinical variables	*N*
Number of patients (females, %)	106 (56, 52.8)
Mean age ± SD (range)	76.4 ± 9.8 (50–95)
Hypertension (%)	84 (79.2)
Diabetes (%)	35 (33.0)
Peripheral artery disease (%)	5 (4.7)
Ischemic heart disease (%)	9 (8.5)
Previous systemic bleeding (%)∗	8 (7.5)
Heart failure (%)∗∗	8 (7.5)
Mean GFR ± SD (range)∗∗∗	72.6 mL/min ± 21.3 (32–122)
GFR < 60 mL/min (%)	29 (27.9)
Median NIHSS (range)	1 (0–18)
Median mRS (range)	1 (0–4)
Median CHADS (range)	4 (2–6)
Median CHA2DS2-VASc (range)	5 (2–9)
CHA2DS2VASc > 5 (%)	75 (70.8)
Median HAS-BLED (range)	2 (1–5)
Reason for starting dabigatran	
Stroke/TIA (%)	101 (95.3)
VKA-related bleeding (%)	5 (4.7)
Previous anticoagulation (%)	39 (36.8)
Ischemic stroke (*n* with INR < 2)	34 (29)
Bleeding (*n* with INR > 2)	5 (5)
Dabigatran dose	
150 mg/12 hours (%)	35 (33)
110 mg/12 hours (%)	71 (67)
Age > 75	66
HAS-BLED ≥ 3	14
GFR 30–50 mL/min	9
Concomitant antiaggregation therapy (%)	9 (8.5)

*Any bleeding with anemia considered as 2-point drop in hemoglobin measured in g/dL and/or need for packed red blood cell transfusion (RCC). ∗∗Congestive heart failure or left ventricular ejection fraction <40. ∗∗∗Measured by Crockcroft-Gault formula. SD: standard deviation, DM: diabetes mellitus, GFR: glomerular filtration rate, NIHSS: National Institutes of Health Stroke Scale, and mRS: Modified Rankin Scale.

**Table 2:** Ischemic complications.

Patient	Age	Baseline mRS	CHA2DS2-VASc	HAS-BLED	Dabigatran dose	Concomitant antiaggregation therapy	Event	TE (months)	Discontinued yes/no
#1	85	3	5	2	110	No	CI	1	Yes
#2	95	1	6	3	110	No	TIA	10	No
#3	79	1	6	3	110	No	CI	1	No
#4	83	1	7	3	110	No	TIA	1	No
#5	50	4	3	1	150	No	TIA	1	No
#6	83	2	6	2	110	No	TIA	1	No
#7	83	2	6	2	110	No	CI∗	1	No
#8	78	0	6	2	110	Yes	TIA	4	No
#9	73	0	6	2	110	No	TIA	6	No
#10	85	3	9	4	110	No	NSTE-ACS	8	No

CI: cerebral infarct. ∗Treated with intravenous tPA. mRS: Modified Rankin Scale. TE: time to event. TIA: transient ischemic attack. NSTE-ACS: non-ST-segment elevation acute coronary syndrome.

**Table 3:** Bleeding complications.

Patient	Age	Baseline mRS	CHA2DS2-VASc	HAS-BLED	Dabigatran dose	Concomitant antiaggregation therapy	Event	TE (months)	Discontinued yes/no	BT
#3	80	1	6	3	110	Yes	UGIB	10	Yes	Yes
#10	85	3	9	4	110	Yes	Rectal bleeding	9	No	No
#11	90	3	6	4	110	No	Rectal bleeding	1	No	No
#12	86	2	5	2	110	No	UGIB	9	Yes	Yes
#12	86	2	5	3	110	No	CH∗∗	9	Yes	No
#13	63	0	3	1	150	No	Metrorrhagia	1	No	No
#14	81	2	6	3	110	No	UGIB	8	Yes	Yes
#15	87	0	5	3	110	No	UGIB	6	No	No
#16	77	3	6	3	110	No	Hematuria	1	No	No
#17	78	0	3	1	110	No	Hematuria	8	No	No

**Administration of prothrombin complex concentrate. mRS: Modified Rankin Scale, TE: time to event, BT: blood transfusion, UGIB: upper gastrointestinal bleeding, and CH: cerebral hemorrhage.
